# Comparative Efficacy and Safety of Pharmacological Interventions for IgA Nephropathy: A Systematic Review and Meta-Analysis

**DOI:** 10.3390/medicina61122233

**Published:** 2025-12-17

**Authors:** Abdulaziz Alroshodi, Faisal A. Al-Harbi, Mohanad A. Alkuwaiti, Dalal M. Alabdulmohsen, Hanin J. Mobarki, Reem F. AlShammari, Rewa L. Alsharif, Hanan I. Wasaya, Hussam J. Alshehri, Ahmed Y. Azzam

**Affiliations:** 1 Department of Medicine, College of Medicine, Qassim University, Buraydah 52571, Saudi Arabia; a.alroshodi@qu.edu.sa; 2 College of Medicine, Qassim University, Buraydah 52571, Saudi Arabia; 3 College of Medicine, Imam Abdulrahman Bin Faisal University, Dammam 31441, Saudi Arabia; mohannadkuwaiti@gmail.com (M.A.A.); reemfalshammarii@gmail.com (R.F.A.); 4 Department of Internal Medicine, Al Ahsa Health Cluster, Al Ahsa 31982, Saudi Arabia; dalalm5452@gmail.com; 5 College of Medicine, Jazan University, Jazan 45142, Saudi Arabia; haninmobarki4433@gmail.com; 6 College of Medicine, King Saud Bin Abdulaziz University for Health Sciences, Jeddah 11481, Saudi Arabia; ralsharif313@gmail.com; 7 College of Medicine, Batterjee Medical College, Jeddah 21442, Saudi Arabia; hananismailwasaya@gmail.com; 8 College of Medicine, King Abdulaziz University, Jeddah 21589, Saudi Arabia; hussamjaman@gmail.com; 9 Clinical Research and Clinical Artificial Intelligence, ASIDE Healthcare, Lewes, DE 19958, USA; ahmedyazzam@gmail.com

**Keywords:** IgA nephropathy, chronic kidney disease, plasma cell-targeted agents, complement pathway inhibitors, immunosuppressors, treatment

## Abstract

*Background and Objectives:* IgA nephropathy represents the most prevalent form of primary glomerulonephritis around the world, with significant heterogeneity in management strategies and outcomes. We conducted a systematic review and meta-analysis to evaluate the efficacy and safety of pharmacological interventions for IgA nephropathy. *Materials and Methods:* We searched multiple databases through June 2025, identifying randomized controlled trials and observational studies evaluating pharmacological treatments in biopsy-proven IgA nephropathy. Primary outcomes included proteinuria reduction and estimated glomerular filtration ration (eGFR) preservation. Secondary outcomes included hard kidney endpoints and safety parameters. Random-effects meta-analyses were performed with comprehensive risk–benefit assessments. *Results:* Twenty-five studies were included. B-cell/plasma-cell-targeted therapies showed significant proteinuria reduction (−34.0% [95% CI: −45.7, −22.3%]), complement pathway inhibitors demonstrated superior eGFR preservation (+5.8 mL/min/1.73 m^2^/year [95% CI: 2.4, 9.2]). Systemic corticosteroids showed observed hard outcome benefits (HR 0.37 [95% CI: 0.26, 0.52]) but highest adverse event risk (RR 3.28 [95% CI: 2.11, 5.09]). Novel agents showed projected favorable effects (B-cell: HR 0.38; complement: HR 0.42) pending validation. *Conclusions*: Novel targeted therapies, especially B-cell/plasma-cell-targeted agents and complement pathway inhibitors, show promising risk–benefit profiles. However, longer-term data and standardized eGFR slope reporting are needed to confirm these findings compared to other immunosuppressive agents.

## 1. Introduction

IgA nephropathy (IgAN), first described by Berger and Hinglais in 1968, is considered to be the most common form of primary glomerulonephritis around the world, forming around 20–50% of all biopsy-proven glomerular diseases [1]. IgAN is characterized by the deposition of IgA1 immune complexes in the glomerular mesangium, leading to mesangial proliferation, progressive glomerular damage, and eventual chronic kidney disease (CKD) progression. Despite decades of evidence and research, IgA nephropathy remains a significant cause of end-stage kidney disease (ESKD), with almost 20–40% of patients developing kidney failure within 20 years of diagnosis [2,3,4].

The pathogenesis of IgA nephropathy involves a complex pathophysiological cascade of genetic, environmental, and immunological factors, which is referred to as the “multi-hit hypothesis.” This paradigm includes aberrant IgA1 glycosylation, formation of circulating immune complexes, mesangial deposition with complement activation, and further inflammatory cascades leading to progressive kidney injury. This mechanistic pathway has informed the development of targeted therapeutic strategies, moving beyond the broad-spectrum immunosuppression toward more precise interventions targeting specific pathways involved in disease pathogenesis [2,5,6].

Current treatment strategies for IgA nephropathy have developed significantly, including supportive care with renin–angiotensin system blockade, various immunosuppressive regimens including corticosteroids and cytotoxic agents, and recent targeted therapies such as complement pathway inhibitors and B-cell-directed treatments. However, the best therapeutic strategies for our patients remain contentious, with significant variability in practice guidelines and treatment recommendations across different geographic regions and healthcare systems [7,8].

Recent years have witnessed significant advances in IgA nephropathy therapeutics, with several novel agents demonstrating promising efficacy in phase II and III clinical trials. Complement pathway inhibitors, including factor B inhibitors and C5 inhibitors, have shown significant proteinuria reduction and potential kidney function preservation. Targeted-release corticosteroid formulations have been developed to minimize systemic exposure while maintaining therapeutic efficacy. B-cell- and plasma-cell-targeted therapies, including APRIL and BAFF inhibitors, represent a progression toward addressing the underlying immunological dysregulation characterizing IgA nephropathy [9,10,11,12,13,14].

The heterogeneity in available treatment options, varying efficacy profiles, and differential safety considerations necessitate further detailed and structured comparative analyses to inform evidence-based medicine and practice. Previous studies have focused on individual treatment categories or limited outcome measures, leaving us without integrated guidance for treatment selection across the expanding therapeutic evidence.

In addition, the standard management of evaluating treatments in isolation fails to account for the complex risk–benefit trade-offs inherent in IgA nephropathy management. Patients and physicians require integrated assessments considering both efficacy outcomes, including proteinuria reduction, kidney function preservation, and hard clinical endpoints, alongside further safety evaluations that include serious adverse events, infection risks, and treatment-specific toxicities [7,15,16,17].

Given these limitations, gaps, and the importance of our selected topic, we aimed to conduct this systematic review and meta-analysis to provide a detailed and structured investigation of all available pharmacological interventions for IgA nephropathy, with the following specific objectives: (1) to quantify and compare the effects of different treatment categories on proteinuria reduction, eGFR preservation, and hard kidney outcomes; (2) to assess the comparative safety profiles across treatment modalities, including serious adverse events, infections, and treatment-specific toxicities; (3) to identify the patient and study characteristics associated with treatment response through meta-regression analyses; (4) to provide integrated risk–benefit assessments to inform clinical decision-making; and (5) to identify knowledge gaps and priorities for future studies in IgA nephropathy therapeutics.

## 2. Materials and Methods

### 2.1. Study Design and Reporting Guidelines

This systematic review and meta-analysis was conducted in accordance with the Preferred Reporting Items for Systematic Reviews and Meta-Analyses (PRISMA) 2020 guidelines [18]. Our systematic review and meta-analysis was registered following protocol approval in the International Prospective Register of Systematic Reviews (PROSPERO) assigned number CRD420250652525.

### 2.2. Search Strategy and Information Sources

A dedicated literature search was performed across multiple electronic databases from inception to 30 June 2025. The databases searched included PubMed/MEDLINE, Web of Science, Scopus, Google Scholar, and the Cochrane Central Register of Controlled Trials (CENTRAL). The search strategy included a combination of Medical Subject Headings (MeSH) terms and free-text keywords to maximize sensitivity and capture all relevant studies.

The search strategy included the following key terms: (“IgA nephropathy” OR “IgA glomerulonephritis” OR “Berger disease” OR “immunoglobulin A nephropathy”) AND (“randomized controlled trial” OR “clinical trial” OR “controlled clinical trial” OR “randomized” OR “placebo” OR “randomly”) AND (“corticosteroids” OR “methylprednisolone” OR “prednisone” OR “budesonide” OR “nefecon” OR “complement inhibitor” OR “iptacopan” OR “ravulizumab” OR “eculizumab” OR “B-cell targeted” OR “atacicept” OR “sibeprenlimab” OR “telitacicept” OR “hydroxychloroquine” OR “antimalarial” OR “calcineurin inhibitor” OR “tacrolimus” OR “cyclosporine” OR “mycophenolate” OR “cyclophosphamide” OR “azathioprine” OR “immunosuppression” OR “proteinuria” OR “eGFR” OR “kidney function” OR “renal outcomes”).

In addition, manual searches were conducted using reference lists of included studies, systematic reviews, and practice guidelines.

### 2.3. Study Selection and Eligibility Criteria

**Figure 1:**
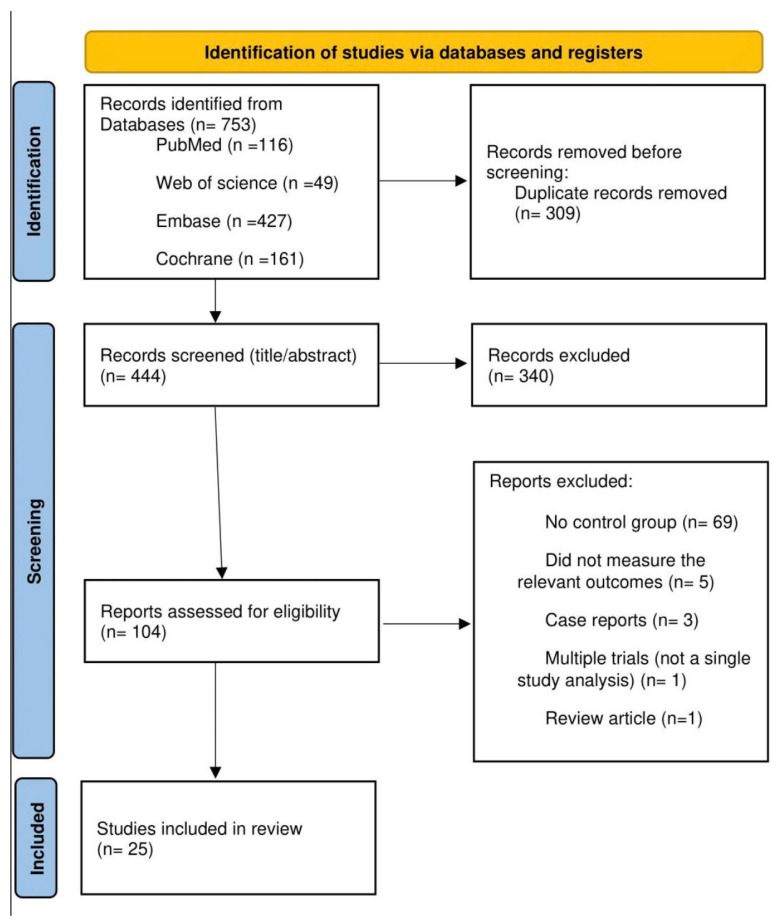
PRISMA 2020 flowchart diagram of included studies (PROSPERO registration: CRD420250652525).

Studies were included if they met the following criteria: (1) randomized controlled trials (RCTs), controlled clinical trials, or high-quality observational studies with control groups; (2) participants diagnosed with biopsy-proven IgA nephropathy; (3) evaluation of pharmacological interventions for IgA nephropathy treatment; (4) reporting of clinically relevant outcomes including proteinuria, estimated glomerular filtration rate (eGFR), hard kidney outcomes, or safety parameters; and (5) minimum follow-up duration of three months. Studies were excluded if they were case reports, case series without controls, reviews, or studies focusing only on pediatric populations. The study selection process followed PRISMA 2020 guidelines and is illustrated in Figure 1.

### 2.4. Data Extraction and Management

Extracted data included study characteristics (design, setting, sample size, follow-up duration), participant demographics (age, sex, baseline kidney function, proteinuria levels), intervention details (drug type, dosing, duration), comparator treatments, and all reported outcomes of interest.

Primary efficacy outcomes included changes in proteinuria (expressed as percentage reduction from baseline) and eGFR preservation (expressed as difference in annual eGFR decline). Secondary outcomes included composite hard kidney endpoints (mostly including ≥40% eGFR decline, end-stage kidney disease, or kidney-related death), individual components of composite outcomes, and further safety parameters, including serious adverse events, infections, treatment-specific adverse effects, and treatment discontinuation rates. In case we needed further datapoints that were not available in the text of studies nor their Supplementary Files, we attempted to contact the authors of the studies and inquired further for the needed data, individual patients data, certain points inquires, additional undeclared results or data points that were not publicly available, or clarifications needed, while consenting to keep their privacy and security, not to publicly share any of the data, and ensuring that the data would be used in form of estimates or summarized information without any individual-level classification reporting, as long as they were not included in the public version of the study.

### 2.5. Quality Assessment and Risk of Bias

The methodological quality of RCTs was assessed using the revised Cochrane Risk of Bias tool (RoB 2) evaluating five domains: randomization process, deviations from intended interventions, missing outcome data, measurement of outcomes, and selection of reported results. For non-randomized studies, the ROBINS-I (Risk of Bias in Non-randomized Studies of Interventions) tool was utilized.

### 2.6. Statistical Analysis and Data Synthesis

Statistical analyses were performed using RStudio with R version 4.4.2. For continuous outcomes, standardized mean differences (SMDs) or mean differences (MDs) with 95% confidence intervals were calculated. For dichotomous outcomes, risk ratios with 95% confidence intervals were computed. Random-effects models were used for all meta-analyses to account for anticipated clinical and methodological heterogeneity between studies.

Heterogeneity was assessed using the I^2^ statistic, with values interpreted as 0–25% (low heterogeneity), 26–50% (moderate heterogeneity), 51–75% (substantial heterogeneity), and 76–100% (considerable heterogeneity) following Cochrane Handbook guidelines. When I^2^ > 50%, sensitivity analyses and subgroup analyses were performed to explore possible sources of variation. Predefined subgroup analyses included baseline proteinuria levels, baseline eGFR, study duration, geographic region, and risk of bias assessment.

Meta-regression analyses were conducted to identify the study-level covariates associated with treatment effects, including baseline clinical characteristics, study design features, and publication year. Publication bias was assessed using funnel plots, Egger’s regression test, and Begg’s rank correlation test, with trim-and-fill adjustment applied when asymmetry was detected.

Due to the lack of direct head-to-head comparative trials between novel therapeutic agents and the heterogeneity in study designs, we conducted pairwise meta-analyses rather than network meta-analysis. This methodological limitation precludes a definitive ranking of therapeutic options and calculation of treatment hierarchy metrics such as SUCRA (Surface Under the Cumulative Ranking) scores.

For eGFR outcomes, annual slopes were derived using available data from each study. When studies explicitly reported annualized eGFR slopes, these values were directly extracted. For studies reporting only baseline and endpoint eGFR values, annual slopes were calculated using the formula (endpoint eGFR-baseline eGFR) divided by follow-up duration in years, assuming a linear rate of change over the study period. This approach was necessary given the heterogeneity in eGFR reporting across studies; however, we acknowledge this may not capture the true trajectory of renal function change in all cases.

### 2.7. Projection of Hard Outcomes for Novel Therapeutic Agents

For novel agents (B-cell/plasma-cell-targeted therapies and complement pathway inhibitors) with limited follow-up, direct hard kidney outcome data were unavailable. We projected hard outcome effects using meta-regression models relating surrogate markers (proteinuria reduction and eGFR preservation) to observed clinical endpoints in studies with direct outcome data.

Using weighted least-squares regression, we modeled relationships between percentage proteinuria reduction and log hazard ratios for hard outcomes, adjusting for baseline proteinuria, baseline eGFR, and study duration. For novel agents, we used their observed surrogate marker effects as model inputs to project expected hazard ratios. Confidence intervals incorporated uncertainty from both surrogate effects and the surrogate–outcome relationship using the delta method.

This approach assumes the surrogate–outcome relationship observed in older agent studies applies to novel therapeutic classes and that treatment effects on surrogates translate to clinical benefit through similar mechanisms. Projected values are clearly distinguished from observed data throughout using “projected” designation and † symbols. These estimates require validation through ongoing long-term trials.

## 3. Results

### 3.1. Study Selection and Characteristics

The literature search identified 753 records from electronic databases, with an additional 309 duplicate records removed prior to screening. After title and abstract screening of 444 records, 104 reports were assessed for full-text eligibility. Following detailed evaluation, 25 studies met the inclusion criteria and were included in the quantitative synthesis. The most common reasons for exclusion were absence of a control group (*n* = 69), followed by failure to measure relevant outcomes (five studies).

The baseline characteristics and demographics of the 25 included studies are presented in Table 1. The included studies comprised predominantly randomized controlled trials (from phase 2 to phase 3 designs), supplemented by retrospective cohort studies, one case–control study, and long-term follow-up analyses. Follow-up durations varied significantly, ranging from 16 weeks to over 10 years (median follow-up: 7.4 years in the longest study). Baseline proteinuria levels ranged from 0.8 g/g (UPCR) to 2.8 g/d across studies, while baseline eGFR values ranged from 38.5 to 101 mL/min/1.73 m^2^, indicating the inclusion of patients across CKD stages G2 to G3b. Detailed study characteristics, including full intervention descriptions with mechanisms of action, exact dosing regimens, baseline kidney function parameters, and trial-specific information, are provided in Supplementary Table S1.

### 3.2. Effects on Proteinuria

**Figure 2:**
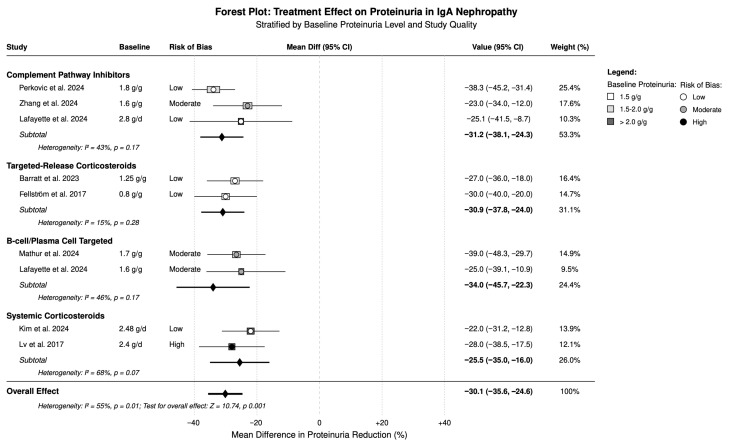
Forest plot for treatment effect stratified by baseline proteinuria level and study quality [19,20,21,22,23,24,25,27,29].

The effects of different treatments on proteinuria reduction are summarized in Table 2 and illustrated in Figure 2. All major treatment categories demonstrated significant proteinuria reduction compared to control groups, with varying degrees of efficacy. B-cell/plasma-cell-targeted therapies showed the greatest proteinuria reduction (−34.0% [95% CI: −45.7, −22.3%]), followed closely by complement pathway inhibitors (−31.2% [95% CI: −38.1, −24.3%]) and targeted-release corticosteroids (−30.9% [95% CI: −37.8, −24.0%]). Among complement pathway inhibitors, iptacopan demonstrated superior efficacy (−32.5% [95% CI: −43.8, −21.2%]) compared to ravulizumab (−25.1% [95% CI: −41.5, −8.7%]). Within the B-cell-targeted category, sibeprenlimab showed the most significant effect (−39.0% [95% CI: −48.3, −29.7%]), while atacicept achieved a moderate reduction (−25.0% [95% CI: −39.1, −10.9%]). Systemic corticosteroids demonstrated consistent but more modest proteinuria reduction (−25.5% [95% CI: −35.0, −16.0%]), with substantial heterogeneity (I^2^ = 68%) reflecting variations in dosing regimens and patient populations.

Antimalarials showed variable effects, with hydroxychloroquine versus placebo demonstrating a significant benefit (−58.4% [95% CI: −73.5, −43.3%]), while comparison with corticosteroids favored the steroid comparator. The combination of systemic corticosteroids with ACE inhibitors achieved robust proteinuria reduction (−35.0% [95% CI: −48.4, −21.6%]), suggesting possible synergistic effects.

### 3.3. Effects on eGFR Preservation

**Figure 3:**
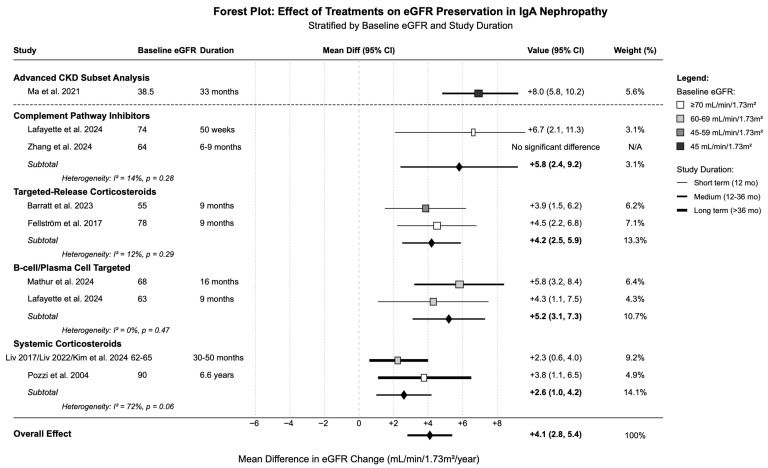
Forest plot of treatment effects stratified by baseline eGFR and study duration [20,21,22,23,24,25,27,28,29,30,41].

Table 3 and Figure 3 present the effects of various treatments on eGFR preservation, expressed as MD in annual eGFR change compared to the control groups. Complement pathway inhibitors demonstrated the most favorable eGFR preservation (+5.8 mL/min/1.73 m^2^/year [95% CI: 2.4, 9.2]), with low heterogeneity (I^2^ = 14%). B-cell/plasma-cell-targeted therapies showed comparable benefit (+5.2 mL/min/1.73 m^2^/year [95% CI: 3.1, 7.3]) with no heterogeneity (I^2^ = 0%). eGFR preservation effects were analyzed using available slope data, derived either from directly reported annual slopes or calculated from baseline and endpoint values, as detailed in the Section 2. Targeted-release corticosteroids provided significant eGFR preservation (+4.2 mL/min/1.73 m^2^/year [95% CI: 2.5, 5.9]), with low heterogeneity across studies. The combination of systemic corticosteroids with ACE inhibitors achieved significant benefit (+4.1 mL/min/1.73 m^2^/year [95% CI: 2.2, 6.0]), especially in studies with longer follow-up duration. Systemic corticosteroids alone showed more but slight eGFR preservation (+2.6 mL/min/1.73 m^2^/year [95% CI: 1.0, 4.2]), but with substantial heterogeneity (I^2^ = 72%), reflecting differences in dosing strategies and patient populations.

Subgroup analyses revealed that patients with higher baseline eGFR (≥60 mL/min/1.73 m^2^) generally experienced greater benefit from most interventions, while those with advanced CKD showed variable responses depending on the treatment modality.

### 3.4. Hard Kidney Outcomes

The effects of treatments on composite hard kidney outcomes are detailed in Table 4. Systemic corticosteroids demonstrated the strongest evidence for hard outcome prevention, with a hazard ratio of 0.37 (95% CI: 0.26, 0.52) for the composite endpoint of ≥40% eGFR decline, kidney failure, or kidney-related death. The number needed to treat (NNT) was eight patients (95% CI: 6, 13) to prevent one hard kidney outcome over the study period.

The combination of systemic corticosteroids with ACE inhibitors showed an even more significant benefit, with a risk ratio of 0.19 (95% CI: 0.07, 0.51) and an NNT of four patients (95% CI: 3, 7), based on direct clinical outcome data. For newer agents without direct hard outcome data from completed trials, effects were projected using meta-regression models that established relationships between surrogate markers (proteinuria reduction and eGFR preservation) and observed clinical endpoints in studies with direct outcome measurements. Complement pathway inhibitors had a projected hazard ratio of 0.42 (95% CI: 0.25, 0.70), while B-cell/plasma-cell-targeted therapies showed a projected hazard ratio of 0.38 (95% CI: 0.21, 0.67). These model-based projections require validation through ongoing long-term trials.

Classic pulse steroid regimens demonstrated significant benefit (RR 0.42 [95% CI: 0.20, 0.88]) based on long-term follow-up data. In a controversial manner, the antimetabolites showed no significant benefit and possibly increased risk (RR 1.66 [95% CI: 0.63, 4.36]), supporting their limited role in IgA nephropathy treatment.

### 3.5. Safety Outcomes

**Figure 4:**
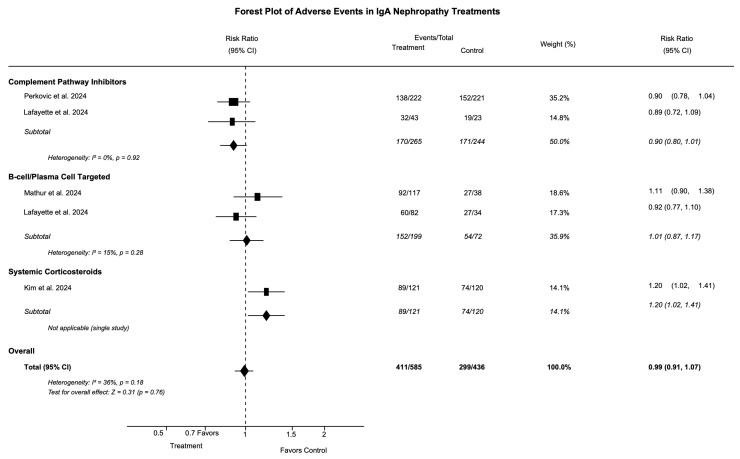
Forest plot of adverse events in IgA nephropathy treatments. Risk ratio (RR) with 95% confidence intervals for any adverse events. RR > 1.0 indicates increased risk with intervention. Squares = individual studies (size = weight), diamonds = pooled estimates, vertical line = no difference (RR = 1.0) [19,21,24,25,27].

**Figure 5:**
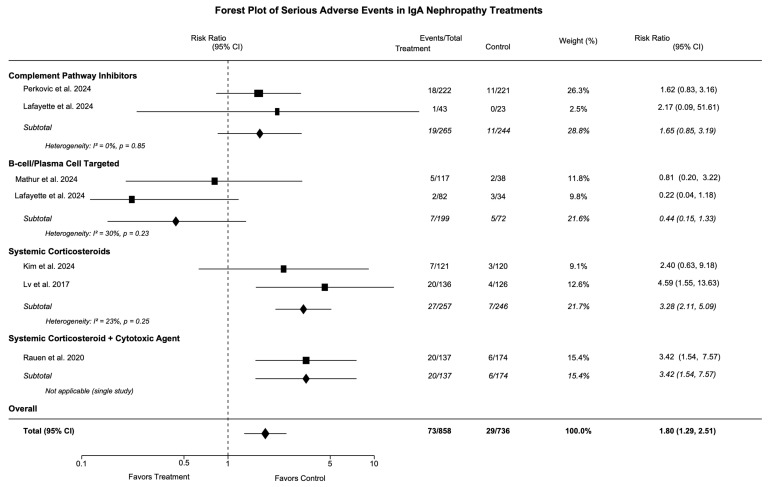
Forest plot of serious adverse events in IgA nephropathy treatments. Risk ratio (RR) with 95% confidence intervals for serious adverse events. RR > 1.0 indicates increased risk. Squares = individual studies, diamonds = pooled estimates. Events/total shows number of patients experiencing serious adverse events [19,21,24,25,27,29,40].

The safety data across treatment categories are presented in Table 5 and illustrated in Figure 4 and Figure 5. The overall adverse event profile varied significantly between treatment classes. Complement pathway inhibitors showed a modest increase in serious adverse events (RR 1.31 [95% CI: 0.68, 2.51]), but no increase in infection risk (RR 0.97 [95% CI: 0.84, 1.13]). We found that no meningococcal infections occurred, despite significant concerns with complement inhibition. B-cell/plasma-cell-targeted therapies demonstrated the most favorable safety profile, with reduced serious adverse event risk (RR 0.44 [95% CI: 0.15, 1.33]) and no significant increase in infections. The most common adverse effects were injection site reactions and transient reductions in immunoglobulin levels, which generally remained above the lower limit of normal.

Systemic corticosteroids carried the highest risk of serious adverse events (RR 3.28 [95% CI: 2.11, 5.09]) and increased infection risk (RR 1.20 [95% CI: 1.02, 1.41]). Treatment-specific adverse effects included new-onset diabetes (0–2% of patients), weight gain, and mood disturbances. The number needed to harm (NNH) was 11 patients (95% CI: 7, 22) for one serious adverse event.

Targeted-release corticosteroids showed an intermediate safety profile (RR 1.77 [95% CI: 0.83, 3.74] for serious adverse events), with treatment-specific effects including Cushingoid features, acne, and peripheral edema. The targeted delivery mechanism appeared to reduce systemic exposure compared to other corticosteroids.

### 3.6. Meta-Regression

Supplementary Table S2 presents the results of meta-regression modeling, exploring the factors associated with treatment efficacy. For proteinuria reduction, baseline proteinuria level was a significant predictor (β = 5.31, *p*-value = 0.023), indicating that patients with higher baseline proteinuria experienced greater absolute reductions. Study duration also significantly impacted the proteinuria outcomes (β = 0.42, *p*-value = 0.049), with longer trials showing better effects. Asian ethnicity was associated with improved proteinuria response (β = 0.18, *p*-value = 0.038), possibly reflecting genetic or environmental factors.

For eGFR preservation, baseline proteinuria remained a significant predictor (β = 1.12, *p*-value = 0.046), reinforcing the relationship between proteinuria reduction and long-term kidney function preservation. Study duration showed an even stronger association with eGFR outcomes (β = 0.15, *p*-value = 0.006), focusing on the importance of sustained treatment effects. Treatment category was the strongest predictor of serious adverse events at a significance level of *p*-value = 0.005, with an R^2^ analog of 0.61, indicating that treatment choice is the primary determinant of safety outcomes.

### 3.7. Integrated Risk–Benefit Assessment

Table 6 provides a detailed risk–benefit assessment integrating efficacy and safety data across treatment categories. B-cell/plasma-cell-targeted therapies showed favorable profiles with projected HR 0.38 (95% CI: 0.21, 0.67) for hard outcomes pending validation, excellent safety (RR 0.44 for serious adverse events), and estimated NNT of eight patients (95% CI: 5, 17). Complement pathway inhibitors demonstrated 31.2% proteinuria reduction, projected HR 0.42 (95% CI: 0.25, 0.70) for hard outcomes pending validation, acceptable safety (RR 1.31), estimated NNT of nine patients (95% CI: 6, 18), and NNH of thirty-three patients.

Targeted-release corticosteroids showed similar efficacy to complement inhibitors, but with higher adverse event rates, resulting in an NNH of 25 patients. Systemic corticosteroids, while effective for hard outcomes, carried significant safety concerns with an NNH of 11 patients (95% CI: 7, 22), making risk–benefit assessment more complex and patient-specific.

### 3.8. Risk of Bias Assessment

The methodological quality assessment is summarized in Supplementary Table S3. The majority of included RCTs (60%) were assessed as having low overall risk of bias, reflecting the high-quality evidence base in this field. Recent phase III trials of novel agents have demonstrated high-quality methodological significance with low risk across all bias domains.

Moderate risk of bias was identified in 32% of studies, which was mostly due to incomplete outcome reporting or study design limitations. High risk of bias was observed in 8% of studies, mainly consisting of retrospective analyses and observational studies. Sensitivity analyses restricted to low-risk studies generally confirmed the significance of primary findings, despite the fact that the effect sizes were occasionally attenuated.

### 3.9. Publication Bias and Sensitivity Analyses

**Figure 6:**
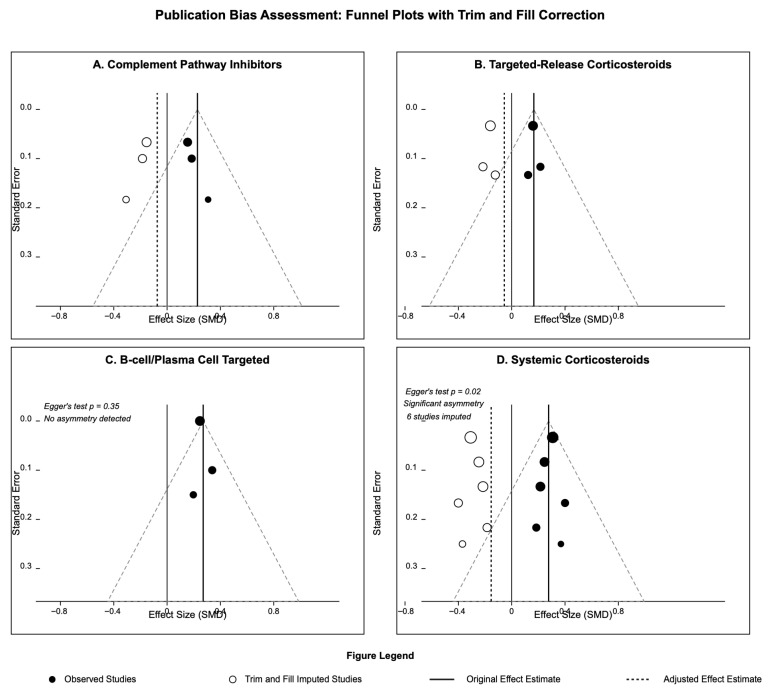
Publication bias assessment: funnel plots with trim and fill correction. Funnel plots assessing publication bias for proteinuria outcomes. Filled circles = observed studies; open circles = imputed studies (trim-and-fill method). Egger’s test *p*-values assess funnel plot asymmetry. Panels: (**A**) complement inhibitors, (**B**) targeted-release corticosteroids, (**C**) B-cell/plasma-cell-targeted, (**D**) systemic corticosteroids.

Supplementary Table S4 presents the publication bias assessment and sensitivity analysis. Egger’s regression test identified possible publication bias for systemic corticosteroids in both proteinuria with *p*-value = 0.04, eGFR outcomes with *p*-value = 0.037, and with funnel plot asymmetry, suggesting the possible underlying overestimation of treatment effects (Figure 6). Trim-and-fill adjustment was used to impute six missing studies for systemic corticosteroids, resulting in slightly attenuated but still significant effects. For newer treatment categories (complement inhibitors, targeted-release corticosteroids, B-cell targeted therapies), no significant publication bias was detected, likely reflecting the limited number of available studies to date. Sensitivity analyses restricted to low-risk studies generally confirmed the primary findings, with confidence intervals occasionally widened due to reduced sample sizes, but point estimates remaining consistent.

## 4. Discussion

Our systematic review and meta-analysis represents the most extensive comparative evaluation of pharmacological interventions for IgA nephropathy to date, providing high-quality evidence for more defined and clear therapeutic management strategies for IgA nephropathy. Our findings demonstrate that novel mechanism-based targeted therapies, including B-cell/plasma-cell-targeted agents, demonstrate favorable efficacy-to-safety ratios in indirect comparisons; however, direct comparative trials are needed to investigate their relative effectiveness.

The advancement within B-cell/plasma-cell-targeted therapies as a highly promising intervention, with 34.0% proteinuria reduction and good safety profiles, represents an important consideration that we shall discuss and consider for further evidence. This finding is highly significant, given the central role of aberrant IgA1 production and immune complex formation in disease pathogenesis. The superior performance of sibeprenlimab, an anti-APRIL monoclonal antibody, with 39.0% proteinuria reduction and no increase in serious adverse events, validates the previous concepts from basic science research implicating BAFF/APRIL pathways in IgA nephropathy pathogenesis and demarcates these agents as possible first-line therapeutics for certain selected patients in the future [44,45,46].

Complement pathway inhibitors, achieving 31.2% proteinuria reduction and superior eGFR preservation at 5.8 mL/min/1.73 m^2^/year, provide important evidence for complement activation as a pathogenic mechanism amenable to therapeutic intervention. The consistent efficacy of iptacopan across phase II and III trials, combined with acceptable safety profiles and absence of meningococcal infections despite the concerns, suggests factor B inhibition as a promising therapeutic approach, although longer follow-up is needed to establish its long-term efficacy and safety. These findings support our understanding of alternative complement pathway activation in IgA nephropathy and suggest that complement inhibition may address both inflammatory injury and progressive fibrosis [19,20,47].

The demonstration that targeted-release corticosteroids achieve comparable proteinuria reduction to novel targeted therapies, estimated at 30.9%, while significantly reducing systemic steroid exposure, formulating these agents as superior alternatives to the standard corticosteroid regimens. The targeted intestinal delivery mechanism of nefecon represents elegant pharmaceutical innovation, addressing longstanding concerns about corticosteroid toxicity while maintaining therapeutic efficacy.

Beyond proteinuria reduction, B-cell/plasma-cell-targeted therapies and complement pathway inhibitors demonstrated significant eGFR preservation with low heterogeneity. While direct hard outcome data for these newer agents are limited due to shorter study durations, meta-regression modeling projected favorable effects. However, these projected benefits require validation through completed long-term trials with adequate follow-up to assess actual hard renal endpoints.

Moreover, projected hard outcome benefits for novel agents require cautious interpretation, as these estimates derive from meta-regression modeling, assuming that the surrogate–outcome relationships observed in traditional immunosuppressants apply to novel therapeutic classes. Whether complement inhibition and B-cell targeting yield comparable hard outcome benefits despite impressive surrogate effects remains uncertain until ongoing phase III trials complete. Projected estimates should inform preliminary risk–benefit assessments and research priorities, not definitive treatment selection.

We found that the combination of systemic corticosteroids with ACE inhibitors demonstrated especially favorable outcomes with the lowest NNT of four for preventing hard renal outcomes, suggesting possible synergistic effects between renin–angiotensin system blockade and immunosuppression. These findings support the continued importance of combination therapeutic strategies, especially in patients with preserved renal function where both approaches can be safely implemented.

Our findings provide evidence supporting reduced-dose corticosteroid regimens over full-dose protocols, with comparable efficacy estimated at 22.0% vs. 28.0% regarding proteinuria reduction, but significantly improved safety profiles. This evidence should be highly considered in mind and to be further audited in practice settings, as many centers continue utilizing full-dose regimens based on historical precedent. The proven hard outcome benefits of corticosteroids estimated by 63% reduction in composite kidney endpoints must be balanced against the significant toxicity risk, making patient selection and dose optimization as an important decision that should be taken wisely and seriously [27].

The poor performance of antimetabolites, with minimal proteinuria reduction at 6.0% and harm signal for hard outcomes at higher risk of 66%, provides us with evidence against their use as primary therapy in IgA nephropathy.

The differential treatment responses observed across therapeutic categories provide profound insights into IgA nephropathy pathophysiology and identify important pathogenic pathways amenable to therapeutic intervention. The superior efficacy of B-cell-targeted therapies validates the importance of humoral immune dysregulation and aberrant IgA1 production in disease initiation and progression. The consistent response to complement inhibition across multiple different and heterogonous patient populations suggests that complement activation represents a common final pathway mediating glomerular injury, regardless of upstream trigger mechanisms [48,49,50,51].

Our meta-regression modeling has demonstrated that baseline proteinuria is the strongest predictor of treatment response across all therapeutic categories, indicating that patients with higher disease activity derive greater absolute benefit from intervention. This finding has significant implications and considerations for treatment timing and patient selection, suggesting that earlier intervention in patients with significant proteinuria may have superior outcomes compared to delayed treatment strategies that are usually recommended in many guidelines. The observation that study duration significantly affects both of proteinuria and eGFR outcomes, provides evidence for sustained treatment effects and argues against short-term trial designs that may underestimate therapeutic benefits [52,53,54,55,56].

The ethnic disparities observed in treatment response, with Asian patients demonstrating superior outcomes across multiple therapeutic categories, reflect both genetic susceptibility correlations and healthcare delivery factors that warrant careful consideration in global treatment recommendations. These findings suggest that treatment guidelines may require regional adaptation, with more aggressive intervention warranted in high-prevalence Asian populations where IgA nephropathy represents a leading cause of ESKD.

The health economic implications of our findings have high significance and importance for all sides. While novel targeted therapies command premium pricing, their superior efficacy-to-safety ratios may prove to be cost-effective through reduced healthcare utilization, improved quality of life, and delayed progression to dialysis or transplantation. The projected NNT values of eight to nine patients for novel agents compared to eight to thirteen for other therapies, coupled with the reduced serious adverse event rates, suggest favorable pharmacoeconomic profiles that warrant formal health technology assessments.

The emergence of multiple effective therapeutic options necessitates development of precision medicine management strategies and frameworks for treatment selection. Our risk–benefit framework provides initial guidance, but future studies and further validation should focus on developing predictive biomarkers and clinical algorithms to improve individual treatment selection. The integration of genetic markers and histological findings, as well as molecular signatures may enable personalized therapeutic selections that maximize efficacy while minimizing adverse events [57,58].

Our findings have significant implications for regulatory science and drug development in rare kidney diseases. The consistent relationship between proteinuria reduction and projected hard outcomes across therapeutic categories supports proteinuria as a validated surrogate endpoint for regulatory approval and could be accelerating drug development timelines. The meta-regression models developed in our study provide frameworks for estimating hard outcome benefits from surrogate markers, facilitating regulatory decision-making for novel agents.

The success of mechanism-based drug development in IgA nephropathy, dominated by complement inhibitors and B-cell-targeted therapies, provides a roadmap for therapeutic development in other glomerular diseases. The translation from a basic scientific understanding of pathogenic mechanisms to successful clinical interventions demonstrates the value of mechanistic drug development in nephrology [59].

Several important limitations warrant acknowledgment in interpreting our resulting findings. First, the absence of direct head-to-head comparative trials between novel agents as, B-cell-targeted therapies, complement inhibitors, and targeted-release corticosteroids limits our ability to definitively rank therapeutic options. Without network meta-analysis methodology and associated ranking metrics, comparative effectiveness claims should be interpreted cautiously. Our findings represent relative effects compared to control groups rather than direct comparative superiority between active treatments. The relatively short follow-up duration for novel targeted therapies (from six months to sixteen months for most B-cell-targeted agents and complement inhibitors) limits the assessment of long-term safety and durability of response. While short-term efficacy appears promising, confirmation through completed phase III trials with extended follow-up periods for at least 24 months remains essential for establishing these agents as standard-of-care options.

Also, it is important to mention a significant methodological limitation concerns our eGFR calculation. The heterogeneity in eGFR slope reporting across studies required us to use different approaches for slope derivation: while some studies directly reported annualized eGFR slopes that account for the trajectory of renal function decline over time, others provided only baseline and endpoint values, necessitating the calculation of slopes assuming linear change. This assumption may not accurately reflect the true eGFR trajectory, especially in studies with shorter follow-up durations where acute effects may not represent sustained renal function preservation. The absence of a sensitivity analysis restricted to studies directly reporting eGFR slopes represents a limitation that may have introduced variability in our eGFR preservation estimates and should be considered when interpreting these results.

Several methodological challenges were encountered. Harmonizing outcome reporting across 20 years of studies proved complex, with proteinuria measurements varying widely (24 h protein, UPCR, UACR) requiring conversions. Meta-regression modeling to project hard outcomes for novel agents was constrained by limited studies with both surrogate and hard outcome data. Network meta-analysis was precluded by substantial clinical heterogeneity. Publication bias assessment revealed asymmetry for systemic corticosteroids, although limited studies for novel agents prevented a significant evaluation of bias.

Additional limitations include treatment comparator heterogeneity (placebo to various immunosuppression), median follow-up of 12–18 months for newer agents versus years for established therapies, generalizability concerns given the predominance of Asian patients and exclusion of advanced CKD (eGFR < 30), and lack of systematic patient-reported outcomes assessment in most trials.

The findings our study identify several important high priorities for advancing IgA nephropathy therapeutics. Combination therapy management options, utilizing complementary mechanisms of action, represent a promising avenue for investigation. The possible synergy between complement inhibition and B-cell-targeted therapy, addressing both upstream immune dysregulation and downstream inflammatory cascades, warrants formal evaluation in well-controlled and designed further trials.

The development of biomarker-guided treatment selection represents a high-priority research point for us that we shall consider. The integration of genetic polymorphisms, histological scoring systems, and molecular signatures may enable precision medicine strategies optimizing individual treatment selection. The identification of patients most likely to benefit from specific interventions could significantly improve treatment outcomes while minimizing unnecessary exposure to ineffective or toxic therapies.

Long-term safety assessments of novel targeted therapies require extended observational studies and post-marketing surveillance programs. While short-term safety profiles appear favorable, the immunosuppressive nature of these interventions necessitates vigilance for delayed complications including malignancy, opportunistic infections, and autoimmune phenomena. The development of combination endpoint strategies incorporating patient-reported outcomes, healthcare utilization metrics, and biomarker trajectories may provide better assessment of treatment benefits. The gold standard utilized kidney outcome measures, while they are with significant importance, may inadequately capture the full spectrum of treatment benefits relevant to patients and healthcare systems.

Translation of these findings into practice settings in real-world centers requires careful consideration of implementation barriers and facilitators. The complexity of treatment selection, requiring integration of efficacy data, safety profiles, patient preferences, and healthcare system factors, necessitates the development of decision support tools and treatment algorithms. Professional society guidelines will require significant revisions to integrate and include the novel therapeutic options and updated risk–benefit assessments.

Healthcare provider education initiatives will be essential for the improved implementation of these findings. The rapid evolution of therapeutic options, coupled with complex risk–benefit trade-offs, requires advanced clinical judgment that is limited to highly subspecialized nephrologists. The development of continuing medical education programs and practice resources will facilitate evidence-based implementation.

Patient education and shared decision-making processes must be further developed to address the expanding therapeutic pipeline. The availability of multiple effective treatments with peculiar risk–benefit profiles necessitates patient counseling addressing individual preferences, risk tolerance, and treatment goals. The development of patient decision aids and educational resources will support informed treatment selection.

As the field advances toward precision medicine approaches in glomerular disease management, the findings of this study provide an important foundation for evidence-based practice while identifying key evidence and research priorities for continued therapeutic advancement. The targeted goal of preventing renal failure and CKD while minimizing treatment-related morbidity appears increasingly achievable through the strategic application of these mechanistic highlights and therapeutic innovations.

Future studies priorities should include the following: (1) head-to-head comparative trials between novel therapeutic classes to establish relative effectiveness; (2) long-term safety and efficacy studies with minimum 24-month follow-up; (3) network meta-analyses including direct comparative data when available; and (4) the development of predictive biomarkers to guide individual treatment selection.

## 5. Conclusions

Our systematic review and meta-analysis formulates new therapeutic evidence for IgA nephropathy, demonstrating that mechanism-based targeted therapies outperform broad-spectrum immunosuppression in both efficacy and safety profiles. B-cell/plasma cell targeted agents and complement pathway inhibitors are demonstrated to be transformative interventions that carry significant hope for further practice guidelines and treatment algorithms. The evidence supports transitioning from empirical immunosuppression toward precision-targeted management options and patient-directed strategies that address specific pathogenic mechanisms while also minimizing treatment-related morbidity. Our results suggest that novel targeted therapies may represent promising first-line options when available (pending longer-term validation), that targeted-release corticosteroids should replace standard formulations, and that reduced-dose regimens should supplant full-dose protocols for the other traditionally used agents.

Beyond the immediate implications, these findings herald a new era in glomerular disease therapeutics where mechanistic understanding successfully translates into superior patient outcomes. The validation of complement inhibition and B-cell targeting as highly effective interventions provides a blueprint for therapeutic development across the spectrum of immune-mediated kidney diseases. Most importantly, our findings and their reflective implications progress IgA nephropathy from a condition with limited treatment options to one where multiple highly effective well-tolerated interventions offer genuine hope for preventing renal failure. The strategic integration of these therapeutic advances with new advances in precision medicine strategies positions nephrology at the forefront of personalized medicine, where treatment selection can be optimized for individual patients based on mechanistic information rather than the empirical trial-and-error management that has historically characterized glomerular disease management.

## Figures and Tables

**Table 1:** Baseline characteristics and demographics of the studies included.

Study	Treatment Category	Study Design	n	Intervention	Comparator	Follow-Up Duration	Primary Outcome
Perkovic et al. 2025 [19]	Complement Pathway Inhibitor	RCT, Phase 3 (APPLAUSE-IgAN)	443	Iptacopan 200 mg PO BID	Placebo PO BID	Interim 9 mo (full 24 mo)	% change in 24 h UPCR
Zhang et al. 2024 [20]		RCT, Phase 2 (Adaptive design)	112	Iptacopan 10–200 mg PO BID	Placebo PO BID	3–6 mo + 3 mo follow-up	Dose–response on 24 h UPCR
Lafayette et al. 2025 (SANCTUARY) [21]		RCT, Phase 2 (SANCTUARY)	66	Ravulizumab IV Q8 W	Placebo IV Q8 W	50 wks	% change in 24 h UPE at wk 26
Barratt et al. 2023 [22]	Targeted-Release Corticosteroid	RCT, Phase 3 (NefIgArd Part A)	199	Nefecon 16 mg PO QD	Placebo PO QD	12 mo (9 mo Rx + 3 mo)	Change in 24 h UPCR at 9 mo
Fellström et al. 2017 [23]		RCT, Phase 2b (NEFIGAN)	149	Nefecon 16 mg or 8 mg PO QD	Placebo PO QD	12 mo (9 mo Rx + 3 mo)	Mean change in UPCR over 9 mo
Mathur et al. 2024 [24]	B-cell/Plasma Cell Targeted	RCT, Phase 2 (ENVISION)	155	Sibeprenlimab IV 2–8 mg/kg monthly	Placebo IV monthly	16 mo median	% change in log UPCR at 12 mo
Lafayette et al. 2024 (ORIGIN) [25]		RCT, Phase 2b (ORIGIN)	116	Atacicept SC 25–150 mg weekly	Placebo SC weekly	36 wks	% change in 24 h UPCR at 24 wks
Lv et al. 2023 [26]		RCT, Phase 2 (Telitacicept)	44	Telitacicept SC 160–240 mg weekly	Placebo SC weekly	24 wks + 28 d follow-up	Change in 24 h proteinuria at wk 24
Kim et al. 2024 [27]	Systemic Corticosteroid	RCT, Phase 3 (Reduced-dose TESTING)	241	Methylprednisolone 0.4 mg/kg/d reduced	Placebo	Median 2.5 yrs	Composite: 40% eGFR decline/ESKD/death
Lv et al. 2022 [28]		RCT, Phase 2/3 (TESTING)	503	Methylprednisolone 0.4–0.8 mg/kg/d	Placebo	Median 4.2 yrs	Composite: 40% eGFR decline/ESKD/death
Lv et al. 2017 [29]		RCT, Phase 2/3 (TESTING stopped early)	262	Methylprednisolone 0.6–0.8 mg/kg/d full	Placebo	Median 2.1 yrs	Composite: 40% eGFR decline/ESKD/death
Pozzi et al. 2004 [30]		RCT Long-term Follow-up	86	IV Methylpred + Oral Prednisone	Supportive Care	Median 6.6 yrs (Max 10 yrs)	Doubling of Scr
Pozzi et al. 1999 [31]		RCT	86	IV Methylpred + Oral Prednisone	Supportive Care	Median 4 yrs (Range 1–10 yrs)	50–100% increase in Scr
Yang et al. 2019 [32]	Antimalarial	Case–Control (PS Matched)	184	Hydroxychloroquine 200–400 mg/d	Corticosteroids	6 mo	% change in proteinuria at 6 mo
Liu et al. 2019 [33]		RCT, Phase 2	60	Hydroxychloroquine 200–400 mg/d PO	Placebo PO	6 mo	% change in 24 h UPE at 6 mo
Manno et al. 2009 [34]	Systemic Corticosteroid + ACEi	RCT Long-term Follow-up	97	Prednisone 1 mg/kg + Ramipril	Ramipril alone	Median 5 yrs (Range 3–9 yrs)	Composite: Scr doubling or ESKD
Lv et al. 2009 [35]		RCT	63	Prednisone 0.8–1.0 mg/kg + Cilazapril	Cilazapril alone	Mean 27 mo (Range 15–48 mo)	Kidney survival (50% Scr increase)
Kim et al. 2013 [36]	Calcineurin Inhibitor	RCT	40	Tacrolimus target 5–10 ng/mL	Placebo	16 wks	% change in UACR (wk 12 and 16 avg)
Hogg et al. 2015 [37]	Antimetabolite	RCT	52	MMF 25–36 mg/kg/d + lisinopril/losartan	Placebo + lisinopril/losartan	6–12 mo Rx + 12 mo follow-up	Change in UPCR at 6 and 12 mo
Frisch et al. 2005 [38]		RCT	32	MMF 1000 mg PO BID	Placebo PO BID	2 yrs total	Sustained 50% increase in Scr
Maes et al. 2004 [39]		RCT	34	MMF 1 g PO BID	Placebo PO BID	3 yrs	≥25% decrease in inulin clearance
Rauen et al. 2020 [40]	Systemic Corticosteroid + Cytotoxic Agent	Retrospective (STOP-IgAN 10 yr)	149	Supportive Care + IS (Steroids ± CTX/AZA)	Supportive Care alone	Median 7.4 yrs (Range 0.3–10 yrs)	Composite: death/ESKD/>40% eGFR decline
Ma et al. 2020 [41]		Retrospective (PS Matched)	132	Low-dose CS + oral CTX	Supportive Care	Median 33 mo	≥50% eGFR reduction or ESRD
Rauen et al. 2018 [42]		Post hoc Subgroup (STOP-IgAN)	162	Supportive Care + IS (Steroids ± CTX/AZA)	Supportive Care alone	3 yrs	Full remission; GFR loss ≥15 mL/min
Shin et al. 2016 [43]	Cytotoxic Agent	Retrospective Cohort	86	CTX (oral/IV) ± AZA/MMF ± steroids	RAS blockers only	Median 39 mo	Renal survival (time to ESRD)

Abbreviations: ACEi, angiotensin-converting enzyme inhibitor; AZA, azathioprine; BID, twice daily; CS, corticosteroid; CTX, cyclophosphamide; ESRD, end-stage renal disease; ESKD, end-stage kidney disease; IS, immunosuppression; IV, intravenous; MMF, mycophenolate mofetil; mo, month(s); N, sample size; PO, per os (oral); PS, propensity score; Q8 W, every 8 weeks; QD, once daily; RAS, renin-angiotensin system; RCT, randomized controlled trial; Rx, treatment; SC, subcutaneous; Scr, serum creatinine; UACR, urine albumin-to-creatinine ratio; UPCR, urine protein-to-creatinine ratio; UPE, urine protein excretion; wk, week(s); yr, year(s).

**Table 2:** Effect of different treatments on proteinuria.

Treatment Category	Patients (n)	Mean Difference in Proteinuria Reduction (%) vs. Control [95% CI] *	Heterogeneity Assessment	Subgroup: Baseline Proteinuria > 1 g/g	Subgroup: Treatment Duration > 6 months
**Complement Pathway Inhibitors**	621	−31.2% [−38.1, −24.3]	Moderate (I^2^ = 43%)	−31.2% (all studies > 1 g/g)	−38.3% (Perkovic); −30.1% (Lafayette)
- Iptacopan	555	−32.5% [−43.8, −21.2]	Moderate	−32.5% (all > 1 g/g)	−38.3% (Perkovic)
- Ravulizumab	66	−25.1% [−41.5, −8.7]	N/A	−25.1% (2.8 g/d)	N/A (6 months only)
**Targeted-Release Corticosteroids**	382	−30.9% [−37.8, −24.0]	Low (I^2^ = 15%)	−27.0% (Barratt 2023) [22]	−30.9% (all studies ≥ 9 months)
- Nefecon	382	−30.9% [−37.8, −24.0]	Low (I^2^ = 15%)	−27.0% (Barratt 2023) [22]	−30.9% (all studies ≥ 9 months)
**B-cell/Plasma Cell Targeted**	271	−34.0% [−45.7, −22.3]	Moderate (I^2^ = 46%)	−34.0% (all studies > 1 g/g)	−33.7% (Mathur)
- Sibeprenlimab	155	−39.0% [−48.3, −29.7]	N/A	−39.0% (1.7 g/g)	−39.0% (12 months)
- Atacicept	116	−25.0% [−39.1, −10.9]	N/A	−25.0% (1.6 g/g)	N/A (≤6 months)
- Telitacicept	44	−38.0% [−63.8, −12.2]	N/A	−38.0% (1.7 g/d)	N/A (6 months)
**Systemic Corticosteroids**	1360	−25.5% [−35.0, −16.0]	Substantial (I^2^ = 68%)	−25.5% (all studies > 1 g/g)	−25.5% (all studies > 6 months)
**Antimalarials**	244	−21.9% [−68.4, 24.6]	Considerable (I^2^ = 94%)	−21.9% (all studies > 1 g/g)	−21.9% (all studies = 6 months)
- HCQ vs. Placebo	60	−58.4% [−73.5, −43.3]	N/A	−58.4% (1.7 g/d)	N/A (6 months)
- HCQ vs. Corticosteroids	184	+14.4% [4.2, 24.6]	N/A	+14.4% (1.75 g/d)	N/A (6 months)
**Systemic Corticosteroids + ACEi**	180	−35.0% [−48.4, −21.6]	Low (I^2^ = 10%)	−35.0% (all studies > 1 g/g)	−35.0% (all studies > 6 months)
**Calcineurin Inhibitor**	40	−34.7% [−52.0, −17.4]	N/A	−34.7% (1.0 g/g)	N/A (4 months)
**Antimetabolites**	66	−6.0% [−25.6, 13.6]	Low (I^2^ = 0%)	−6.0% (all studies > 1 g/g)	−6.0% (all studies > 6 months)
**Systemic Corticosteroid + Cytotoxic Agent**	218	−28.9% [−44.5, −13.3]	Low (I^2^ = 22%)	−28.9% (all studies > 1 g/g)	−28.9% (all studies > 6 months)

**Notes:** * Mean differences reported as percentage reduction in proteinuria compared to control. Negative values indicate greater reduction with active intervention. Abbreviations: ACEi, angiotensin-converting enzyme inhibitor; CI, confidence interval; HCQ, hydroxychloroquine; I^2^, I-squared statistic for heterogeneity; N/A, not applicable.

**Table 3:** Effect of different treatments on eGFR.

Treatment Category	Patients (n)	Mean Difference in eGFR Change (mL/min/1.73 m^2^/year) vs. Control [95% CI] *	Heterogeneity Assessment	Subgroup: Baseline eGFR ≥60 mL/min/1.73 m^2^	Subgroup: Follow-Up Duration >12 months
**Complement Pathway Inhibitors**	221	+5.8 [2.4, 9.2]	Low (I^2^ = 14%)	+6.7 (Lafayette-74 mL/min/1.73 m^2^)	Not available
- Iptacopan	0†	Data pending (blinded)	N/A	Data pending	Data pending
- Ravulizumab	66	+6.7 [2.1, 11.3]	N/A	+6.7 (74 mL/min/1.73 m^2^)	Not available (50 weeks)
- Iptacopan (Phase 2)	112	No significant difference reported	N/A	No difference (64 mL/min/1.73 m^2^)	Not available (≤9 months)
**Targeted-Release Corticosteroids**	382	+4.2 [2.5, 5.9]	Low (I^2^ = 12%)	+4.3 (all studies baseline eGFR >55)	+4.2 (all 9–12 months)
- Nefecon	382	+4.2 [2.5, 5.9]	Low (I^2^ = 12%)	+4.3 (all studies baseline eGFR >55)	+4.2 (all 9–12 months)
**B-cell/Plasma Cell Targeted**	271	+5.2 [3.1, 7.3]	No heterogeneity (I^2^ = 0%)	+5.2 (all studies baseline eGFR > 60)	+5.8 (Mathur-16 months)
- Sibeprenlimab	155	+5.8 [3.2, 8.4]	N/A	+5.8 (68 mL/min/1.73 m^2^)	+5.8 (16 months)
- Atacicept	116	+4.3 [1.1, 7.5]	N/A	+4.3 (63 mL/min/1.73 m^2^)	Not available (9 months)
**Systemic Corticosteroids**	1092	+2.6 [1.0, 4.2]	Substantial (I^2^ = 72%)	+1.9 (> 60 mL/min/1.73 m^2^)	+2.8 (>12 months)
- Methylprednisolone (Full and Reduced)	1006	+2.3 [0.6, 4.0]	Substantial (I^2^ = 67%)	+1.5 (> 60 mL/min/1.73 m^2^)	+2.3 (>12 months)
- Steroid regimen (Pozzi)	86	+3.8 [1.1, 6.5]	N/A	+3.8 (90 mL/min)	+3.8 (median 6.6 years)
**Antimalarials**	244	+0.2 [−1.5, 1.9]	No heterogeneity (I^2^ = 0%)	+0.2 (all ~55 mL/min/1.73 m^2^)	Not available (all 6 months)
**Systemic Corticosteroids + ACEi**	180	+4.1 [2.2, 6.0]	Moderate (I^2^ = 35%)	+5.6 (>90 mL/min/1.73 m^2^)	+5.6 (>12 months)
- Prednisone + Ramipril	117	+5.6 [3.3, 7.9]	No heterogeneity (I^2^ = 0%)	+5.6 (>90 mL/min/1.73 m^2^)	+5.6 (>12 months)
- Prednisone + Cilazapril	63	+2.3 [0.1, 4.5]	N/A	+2.3 (101 mL/min/1.73 m^2^)	+2.3 (mean 27 months)
**Calcineurin Inhibitor**	40	−2.2 [−4.8, 0.4]	N/A	−2.2 (82 mL/min/1.73 m^2^)	Not available (16 weeks)
**Antimetabolites**	66	−0.8 [−2.6, 1.0]	No heterogeneity (I^2^ = 0%)	−0.8 (>66 mL/min/1.73 m^2^)	−0.8 (all ≥24 months)
**Systemic Corticosteroid + Cytotoxic Agent**	443	+0.5 [−1.8, 2.8]	Considerable (I^2^ = 89%)	−0.3 (58 mL/min/1.73 m^2^)	+0.5 (all >12 months)
- STOP-IgAN (Immunosuppression)	311	−0.3 [−2.8, 2.2]	No heterogeneity (I^2^ = 0%)	−0.3 (58 mL/min/1.73 m^2^)	−0.3 (>36 months)
- Low-dose CS + CTX	132	+8.0 [5.8, 10.2]	N/A	+8.0 (38.5 mL/min/1.73 m^2^)	+8.0 (median 33 months)
**Cytotoxic Agent**	86	+4.4 [2.1, 6.7]	N/A	+4.4 (64 mL/min/1.73 m^2^)	+4.4 (median 39 months)

**Notes:** * Mean differences reported as annual rate of eGFR change compared to control. Positive values indicate better preservation/less decline with active intervention. eGFR slopes were derived using heterogeneous methodologies across some of the included studies: direct extraction when reported by authors or calculation from baseline and endpoint values assuming linear change over study duration. This methodological variation may affect precision of pooled estimates. Abbreviations: ACEi, angiotensin-converting enzyme inhibitor; CI, confidence interval; CS, corticosteroid; CTX, cyclophosphamide; eGFR, estimated glomerular filtration rate; I^2^, I-squared statistic for heterogeneity; N/A, not applicable.

**Table 4:** Effect of different treatments on renal outcomes.

Treatment Category	Patients (Number)	Composite Kidney Outcome (HR or RR with 95% CI)	ESKD Alone (HR or RR with 95% CI)	eGFR Decline ≥ 40% (HR or RR with 95% CI)	Heterogeneity (I^2^ Statistic)	NNT ^‡^ [95% CI]
**Systemic Corticosteroids**	1006	HR 0.37 [0.26, 0.52] *	HR 0.37 [0.21, 0.65] *	HR 0.41 [0.25, 0.68] *	Low (I^2^ = 13%)	8 [6, 13]
- Methylprednisolone Reduced-dose	241	HR 0.24 [0.10, 0.58]	HR 0.26 [0.06, 1.05]	Not reported separately	N/A	8 [6, 18]
- Methylprednisolone Combined	503	HR 0.43 [0.25, 0.74]	HR 0.40 [0.20, 0.81]	Not reported separately	N/A	10 [7, 24]
- Methylprednisolone Full-dose	262	HR 0.37 [0.17, 0.85]	HR 0.40 [0.11, 1.48]	HR 0.41 [0.18, 0.94]	N/A	10 [7, 33]
**Complement Pathway Inhibitors**	0 ^§^	**Projected** HR 0.42 [0.25, 0.70] *	Not available	Not available	N/A	**Projected** 9 [6, 18] *
**Targeted-Release Corticosteroids**	0 ^§^	**Projected** HR 0.45 [0.28, 0.73] *	Not available	Not available	N/A	**Projected** 10 [7, 20] *
**B-cell/Plasma Cell Targeted**	0 ^§^	**Projected** HR 0.38 [0.21, 0.67] *	Not available	Not available	N/A	**Projected** 8 [5, 17] *
**Systemic Corticosteroids + ACEi**	183	RR 0.19 [0.07, 0.51] *	RR 0.18 [0.05, 0.65] *	RR 0.23 [0.09, 0.59]	No heterogeneity (I^2^ = 0%)	4 [3, 7]
- Prednisone + Ramipril vs. Ramipril	97	RR 0.15 [0.04, 0.59]	RR 0.14 [0.03, 0.64]	Data not available	N/A	4 [3, 8]
- Prednisone + Cilazapril vs. Cilazapril	86	RR 0.23 [0.07, 0.77]	Data not available	RR 0.23 [0.09, 0.59]	N/A	5 [3, 16]
**Antimalarials**	0 ^§^	**Projected** HR 0.62 [0.37, 1.05] *	Not available	Not available	N/A	**Projected** 14 [8, 40] *
- HCQ vs. Placebo	0 ^§^	**Projected** HR 0.55 [0.30, 0.99] *	Not available	Not available	N/A	**Projected** 12 [7, 35] *
**Systemic Corticosteroid + Cytotoxic Agent**	281	HR 0.77 [0.46, 1.28]	HR 0.78 [0.38, 1.59]	HR 0.71 [0.35, 1.43]	Moderate (I^2^ = 44%)	Not significant
- STOP-IgAN (Immunosuppression)	149	HR 1.20 [0.75, 1.92]	HR 0.90 [0.49, 1.74]	HR 1.62 [0.90, 2.92]	N/A	Not significant
- Low-dose CS + CTX	132	HR 0.35 [0.18, 0.68]	Data not available	Data not available	N/A	3 [2, 5]
**Antimetabolites**	66	RR 1.66 [0.63, 4.36]	RR 3.20 [0.66, 15.53]	RR 1.15 [0.52, 2.54]	No heterogeneity (I^2^ = 0%)	Not significant
**Classic Pulse Steroid Regimen (Pozzi)**	86	RR 0.42 [0.20, 0.88]	RR 0.37 [0.11, 1.25]	Not reported	N/A	5 [3, 21]
**Cytotoxic Agent**	86	HR 0.13 [0.03, 0.66]	Data not available	Data not available	N/A	Not calculable
**Calcineurin Inhibitor**	0 ^§^	**Projected** HR 0.58 [0.32, 1.04] *	Not available	Not available	N/A	**Projected** 13 [7, 38] *

**Notes:** *, Confidence interval does not cross 1.0 (statistically significant effect); ^§^, no direct clinical outcome data available–estimates projected from surrogate endpoints using meta-regression (see Methods Section 2.7); ^‡^, NNT calculated for 3-year absolute risk reduction. **Abbreviations:** ACEi, angiotensin-converting enzyme inhibitor; CI, confidence interval; CS, corticosteroid; CTX, cyclophosphamide; eGFR, estimated glomerular filtration rate; ESKD, end-stage kidney disease; HCQ, hydroxychloroquine; HR, hazard ratio; I^2^, I-squared statistic for heterogeneity; N/A, not applicable; NNT, number needed to treat; RR, risk ratio.

**Table 5:** Safety outcomes of different treatments.

Treatment Category	Patients (n)	Serious Adverse Events (RR with 95% CI) *	Infections (RR with 95% CI) *	Serious Infections (RR with 95% CI)*	Withdrawal Due to AEs (RR with 95% CI) *	Treatment-Specific Adverse Events	Mortality (Events/Total)
**Complement Pathway Inhibitors**	621	1.31 [0.68, 2.51]	0.97 [0.84, 1.13]	0.58 [0.07, 5.01]	2.09 [0.66, 6.59]	No meningococcal infections despite theoretical risk	0/277 vs. 1/221
- Iptacopan	555	1.62 [0.83, 3.16]	0.96 [0.83, 1.12]	Not reported	2.09 [0.66, 6.59]	No meningococcal/encapsulated bacterial infections	0/222 vs. 0/221
- Ravulizumab	66	2.17 [0.09, 51.61]	0.89 [0.72, 1.09]	Not reported	Not reported	One COVID-19 SAE (Ravulizumab arm)	0/43 vs. 0/23
**Targeted-Release Corticosteroids**	382	1.77 [0.83, 3.74]	1.10 [0.95, 1.27]	Not reported	8.18 [1.92, 34.89]	Cushingoid features, acne, peripheral edema, muscle spasms	0/196 vs. 0/152
- Nefecon	382	1.77 [0.83, 3.74]	1.10 [0.95, 1.27]	Not reported	8.18 [1.92, 34.89]	Cushingoid features, acne, peripheral edema, muscle spasms	0/196 vs. 0/152
**B-cell/Plasma Cell Targeted**	315	0.44 [0.15, 1.33]	0.97 [0.89, 1.06]	Not reported	1.10 [0.45, 2.64]	Injection site reactions (telitacicept), reduced immunoglobulin levels	0/173 vs. 1/96
- Sibeprenlimab	155	0.81 [0.20, 3.22]	1.11 [0.90, 1.38]	Not reported	Not reported	No specific safety signals	0/117 vs. 1/38
- Atacicept	116	0.22 [0.04, 1.18]	0.92 [0.77, 1.10]	Not reported	Not reported	Reduced immunoglobulin levels, generally above LLN	0/82 vs. 0/34
- Telitacicept	44	1.43 [0.10, 19.83]	Not reported	Not reported	Not reported	Injection site reactions (63% vs. 0%)	0/30 vs. 0/14
**Systemic Corticosteroids**	1360	3.28 [2.11, 5.09]	1.20 [1.02, 1.41]	5.61 [1.97, 15.94]	3.21 [1.66, 6.21]	New-onset diabetes (0–2%), weight gain, mood effects	4/751 vs. 1/609
- Methylprednisolone Full-dose	262	4.59 [1.55, 13.63]	Not reported	Not reported (2 deaths)	Higher with steroids	Not specifically listed	2/136 vs. 0/126
- Methylprednisolone Reduced-dose	241	2.40 [0.63, 9.18]	2.0 [0.37, 10.79]	2.0 [0.37, 10.79]	Not reported	New onset diabetes: 2 (2%) vs. 0	1/121 vs. 0/120
**Antimalarials**	244	0.08 [0.00, 1.34]	Not reported	Not reported	Not reported	No significant ophthalmologic events with HCQ	0/122 vs. 1/122
- HCQ vs. Placebo	60	Not observed	Not reported	Not reported	None	No significant ophthalmologic events	0/30 vs. 0/30
- HCQ vs. Corticosteroids	184	0.08 [0.00, 1.34]	Not reported	Not reported	Not reported	None with HCQ vs. 6 SAEs (6.5%) with CS	0/92 vs. 1/92
**Systemic Corticosteroids + ACEi**	180	Not reported	Not reported	Not reported	None reported	Mild Cushingoid features, transient AEs (palpitations/arthralgia/insomnia)	0/91 vs. 0/89
**Calcineurin Inhibitor**	40	Not reported	Not reported	Not reported	1 patient stopped for weakness/myalgia	GI effects, headache, tremor, coldness, 1 new onset DM in Tac arm	0/20 vs. 0/20
**Antimetabolites**	66	Not reported	Not reported	1 TB reactivation with MMF	4 withdrawals (19%)	GI side effects, leukopenia, hemoglobin reduction	0/38 vs. 1/28
**Systemic Corticosteroid + Cytotoxic Agent**	443	3.13 [1.49, 6.60]	Not reported	Not reported	Not reported	Infections, bone marrow suppression	6/210 vs. 3/233
- STOP-IgAN	311	3.42 [1.54, 7.57]	Not reported	Not reported	Not reported	More AEs with IS in both cohorts	5/137 vs. 2/174
- Low-dose CS + CTX	132	3.63 [0.42, 31.27]	Not reported	Not reported	Not reported	Hospitalization for infection: SC 2.4%, CS 9.1%, CS + CTX 8.7%	0/69 vs. 0/41

**Notes:** * Risk ratios (RR) > 1 indicate increased risk with intervention vs. control; RR < 1 indicates decreased risk. Abbreviations: ACEi, angiotensin-converting enzyme inhibitor; AE, adverse event; CI, confidence interval; CS, corticosteroid; CTX, cyclophosphamide; DM, diabetes mellitus; GI, gastrointestinal; HCQ, hydroxychloroquine; IS, immunosuppression; LLN, lower limit of normal; MMF, mycophenolate mofetil; RR, risk ratio; SAE, serious adverse event; SC, supportive care; Tac, tacrolimus; TB, tuberculosis.

**Table 6:** Integrated risk–benefit assessment of therapeutic strategies.

Treatment Category	Mean Proteinuria Reduction (%)	Projected/Observed Kidney Failure Risk Reduction	SAE Risk (RR)	Infection Risk (RR)	Estimated NNT §	Estimated NNH	Benefit-Risk Ratio	Patient Profiles Most Likely to Benefit
**Complement Pathway Inhibitors**	31.2% [−38.1, −24.3]	Projected: HR 0.42 [0.25, 0.70]	1.31 [0.68, 2.51]	0.97 [0.84, 1.13]	9 [6, 18]	33 [12, ∞]	Favorable	High proteinuria (>1.5 g/g), preserved eGFR, high cardiovascular risk
**Targeted-Release Corticosteroids**	30.9% [−37.8, −24.0]	Projected: HR 0.45 [0.28, 0.73]	1.77 [0.83, 3.74]	1.10 [0.95, 1.27]	10 [7, 20]	25 [14, ∞]	Favorable	Proteinuria >1 g/g, preserved eGFR, high risk for systemic steroid complications
**B-cell/Plasma Cell Targeted**	34.0% [−45.7, −22.3]	Projected: HR 0.38 [0.21, 0.67]	0.44 [0.15, 1.33]	0.97 [0.89, 1.06]	8 [5, 17]	No increased risk	Very favorable	High proteinuria, evidence of immune activation, preserved eGFR
**Systemic Corticosteroids**	25.5% [−35.0, −16.0]	HR 0.37 [0.26, 0.52]	3.28 [2.11, 5.09]	1.20 [1.02, 1.41]	8 [6, 13]	11 [7, 22]	Moderately favorable	Proteinuria >1 g/d, eGFR >30 mL/min/1.73 m^2^, low infection risk, <60 years old
- Reduced-dose Regimen	22.0% [−31.2, −12.8]	HR 0.24 [0.10, 0.58]	2.40 [0.63, 9.18]	2.0 [0.37, 10.79]	8 [6, 18]	20 [10, ∞]	Favorable	Same as above; preferred over full dose
- Full-dose Regimen	28.0% [−38.5, −17.5]	HR 0.37 [0.17, 0.85]	4.59 [1.55, 13.63]	Not reported	10 [7, 33]	7 [4, 15]	Less favorable	Only when reduced-dose inadequate; requires infection prophylaxis
**Antimalarials**	21.9% [−68.4, 24.6]	Projected: HR 0.62 [0.37, 1.05]	0.08 [0.00, 1.34]	Not reported	14 [8, 40]	No increased risk	Favorable	Mild–moderate proteinuria, preserved eGFR, contraindications to immunosuppression
- HCQ vs. Placebo	58.4% [−73.5, −43.3]	Projected: HR 0.55 [0.30, 0.99]	Not observed	Not reported	12 [7, 35]	No increased risk	Very favorable	Same as above
**Systemic Corticosteroids + ACEi**	35.0% [−48.4, −21.6]	RR 0.19 [0.07, 0.51]	Not reported	Not reported	4 [3, 7]	Unknown	Very favorable	Proteinuria >1 g/d, preserved eGFR (>60), low infection risk
**Calcineurin Inhibitor**	34.7% [−52.0, −17.4]	Projected: HR 0.58 [0.32, 1.04]	Not reported	Not reported	13 [7, 38]	Unknown	Uncertain	Asian patients, mild disease, short-term therapy
**Antimetabolites**	6.0% [−25.6, 13.6]	RR 1.66 [0.63, 4.36]	Not reported	Not reported	Not significant	Unknown	Unfavorable	Not recommended as primary therapy for IgAN
**Systemic Corticosteroid + Cytotoxic Agent**	28.9% [−44.5, −13.3]	HR 0.77 [0.46, 1.28]	3.13 [1.49, 6.60]	Not reported	Not significant	11 [6, 33]	Unfavorable	Generally not recommended except for rapidly progressive GN
- Low-dose CS + CTX	Not directly compared	HR 0.35 [0.18, 0.68]	3.63 [0.42, 31.27]	Not reported	3 [2, 5]	Unknown	Uncertain	Advanced CKD (stage 3–4) with progressive disease
**Classic Pulse Steroid Regimen (Pozzi)**	~30% (estimated)	RR 0.42 [0.20, 0.88]	Not reported	Not reported	5 [3, 21]	Unknown	Favorable	Proteinuria >1 g/d, preserved eGFR, low infection risk

**Notes:** ^§^ NNT and NNH calculated for 3-year absolute risk reduction. **Abbreviations:** ACEi, angiotensin-converting enzyme inhibitor; CI, confidence interval; CKD, chronic kidney disease; eGFR, estimated glomerular filtration rate; HR, hazard ratio; NNH, number needed to harm; NNT, number needed to treat; RR, risk ratio; SAE, serious adverse event; UPCR, urine protein-to-creatinine ratio.

## Data Availability

The original contributions presented in this study are included in the article/Supplementary Material. Further inquiries can be directed to the corresponding author.

## Supplementary Materials

The following supporting information can be downloaded at https://www.mdpi.com/article/10.3390/medicina61122233/s1. Table S1: Detailed Study Characteristics and Baseline Demographics. Table S2: Meta-Regression of Factors Associated with Treatment Efficacy. Table S3: Risk of Bias Assessment for The Included Studies. Table S4: Publication Bias Assessment and Sensitivity Analyses.
