# Supplementary material for: Comparative Efficacy and Safety of Pharmacological Interventions for IgA Nephropathy: A Systematic Review and Meta-Analysis
Source: Medicina (Kaunas). 2025 Dec 17;61(12):2233. doi: 10.3390/medicina61122233 (PMC12734590; doi:10.3390/medicina61122233)
Supplement: Supplementary file 1 [file medicina-61-02233-s001.zip › medicina-3983870-supplementary.pdf]

Supplementary Table S1: Detailed Study Characteristics and Baseline Demographics

Study	Detailed Intervention Description	Detailed Comparator Description	Baseline Proteinuria	Baseline eGFR (mL/min/1.73m²)	Additional Trial Details
Perkovic et al. 2024	Iptacopan (Factor B inhibitor) 200 mg PO BID for 24 months	Placebo PO BID for 24 months	UPCR ~1.8 g/g	~64	APPLAUSE-IgAN trial; Phase 3; interim analysis at 9 months reported; full study duration 24 months
Zhang et al. 2024	Iptacopan (Factor B inhibitor) 10, 50, 100, or 200 mg PO BID for 3 or 6 months	Placebo PO BID for 3 or 6 months	UPCR ~1.6 g/g	~64	Phase 2 adaptive design trial; 3 or 6 months treatment plus 3 months follow-up
Lafayette et al. 2024 (SANCTUARY - Ravulizumab)	Ravulizumab (C5 inhibitor) IV weight-based dosing Q8W for 26 weeks (double-blind), then open-label ravulizumab to week 50	Placebo IV Q8W for 26 weeks, then open-label ravulizumab	2.8 g/d	~74	SANCTUARY trial; Phase 2; double-blind phase 26 weeks, then open-label extension to week 50
Barratt et al. 2023	Nefecon (targeted-release budesonide) 16 mg PO QD for 9 months	Placebo PO QD for 9 months	UPCR ~1.25 g/g	~55	NefIgArd Part A trial; Phase 3; 9 months treatment followed by 3 months follow-up (12 months total)
Fellström et al. 2017	Nefecon (targeted-release budesonide) 16 mg or 8 mg PO QD for 9 months	Placebo PO QD for 9 months	UPCR 0.8 g/g	78	NEFIGAN trial; Phase 2b; 9 months treatment followed by 3 months follow-up (12 months total)
Mathur et al. 2024	Sibeprenlimab (anti-APRIL monoclonal antibody) IV 2, 4, or 8 mg/kg monthly for 12 months	Placebo IV monthly for 12 months	UPCR ~1.7 g/g	~68	ENVISION trial; Phase 2; 16 months median follow-up; dose-ranging study
Lafayette et al. 2024 (ORIGIN - Atacicept)	Atacicept (anti-BAFF/APRIL fusion protein) SC 25, 75, or 150 mg weekly for 36 weeks	Placebo SC weekly for 36 weeks	UPCR 1.6 g/g	63	ORIGIN trial; Phase 2b; 36 weeks double-blind treatment; dose-ranging study
Lv et al. 2023	Telitacicept (anti-BLyS/APRIL fusion protein) SC 160 mg or 240 mg weekly for 24 weeks	Placebo SC weekly for 24 weeks	~1.86 g/d	~79	Phase 2 trial; 24 weeks treatment followed by 28-day follow-up; dose-ranging study
Kim et al. 2024	Reduced-dose oral methylprednisolone (0.4 mg/kg/d, maximum 32 mg/d) for 2 months, then tapered over 4-7 months	Placebo for 6-9 months	2.48 g/d	65	Reduced-dose TESTING trial; Phase 3; median 2.5 years follow-up
Lv et al. 2022	Oral methylprednisolone (full-dose: 0.6-0.8 mg/kg/d tapered; reduced-dose: 0.4 mg/kg/d tapered) for 6-9 months total	Placebo for 6-9 months	2.4 g/d	~62	Full and Reduced-dose TESTING trial combined; Phase 2/3; median 4.2 years follow-up
Lv et al. 2017	Full-dose oral methylprednisolone (0.6-0.8 mg/kg/d, maximum 48 mg/d) for 2 months, then tapered over 4-6 months	Placebo for 6-8 months	2.4 g/d	~59	Full-dose TESTING trial stopped early; Phase 2/3; median 2.1 years follow-up
Pozzi et al. 2004	Original RCT: Steroids (IV Methylprednisolone 1g ×3 days at months 1, 3, 5 + Oral Prednisone 0.5 mg/kg alternate day for 6 months)	Original RCT: Supportive Care alone	1.9 g/d	~90 (CrCl)	Long-term follow-up study; median 6.6 years (maximum ~10 years); creatinine clearance reported
Pozzi et al. 1999	Steroids (IV Methylprednisolone 1g ×3 days at months 1, 3, 5 + Oral Prednisone 0.5 mg/kg alternate day for 6 months)	Supportive Care alone	1.9 g/d	~90 (CrCl)	Median 4 years follow-up (range 1-10 years); creatinine clearance reported
Yang et al. 2019	Hydroxychloroquine (HCQ) dose adjusted by eGFR (typically 200-400 mg/d) for 6 months	Corticosteroids (prednisone equivalent, various regimens) for 6-8 months	1.75 g/d	~56	Case-control study with propensity score matching; 6 months follow-up
Liu et al. 2019	Hydroxychloroquine (HCQ) dose adjusted by eGFR (200-400 mg/d) PO daily for 6 months	Placebo PO daily for 6 months	1.7 g/d	~54	Phase 2 RCT; 6 months treatment and follow-up
Manno et al. 2009	Original RCT: Prednisone (1 mg/kg tapered over 6 months) + Ramipril	Original RCT: Ramipril alone	1.6 g/d	~99	Long-term follow-up study; median 5 years (range 3-9 years)
Lv et al. 2009	Prednisone (0.8-1.0 mg/kg tapered over 6-8 months) + Cilazapril	Cilazapril alone	2.2 g/d	~101	RCT; mean ~27 months follow-up (range 15-48 months)
Kim et al. 2013	Tacrolimus PO dose adjusted to trough 5-10 ng/mL for 8 weeks, then 0.05 mg/kg/d	Placebo PO for 16 weeks	UACR ~1.0 g/g	~82	Phase 2 RCT; 16 weeks treatment; primary outcome averaged weeks 12 and 16
Hogg et al. 2015	Mycophenolate mofetil (MMF) target dose 25-36 mg/kg/d in addition to lisinopril/losartan + Omacor	Placebo in addition to lisinopril/losartan + Omacor	UPCR ~1.5 g/g	~100	RCT; 6-12 months treatment plus 12 months follow-up; all patients received background RAS blockade
Frisch et al. 2005	Mycophenolate mofetil (MMF) 1000 mg PO BID for 1 year	Placebo PO BID for 1 year	2.7 g/d	~66 (CrCl)	RCT; 2 years total follow-up; creatinine clearance reported
Maes et al. 2004	Mycophenolate mofetil (MMF) 1 g PO BID for 3 years	Placebo PO BID for 3 years	1.6 g/d	~71 (inulin clearance)	RCT; 3 years treatment and follow-up; inulin clearance used as GFR measure
Rauen et al. 2020	Original RCT: Supportive Care + Immunosuppression (Steroids if baseline eGFR≥60; Steroids + Cyclophosphamide/Azathioprine if baseline eGFR 30-59) for 6 months or 3 years	Original RCT: Supportive Care alone	1.1 g/d	~58	STOP-IgAN 10-year retrospective long-term follow-up; median 7.4 years (range 0.3-10 years)
Ma et al. 2020	Low-dose Corticosteroids (Prednisone ~30 mg/d for 3 months, then tapered) + oral Cyclophosphamide (50 mg/d for 5 months)	Uncontrolled Supportive Care	1.9 g/d	38.5	Retrospective cohort with propensity score matched subset; median 33 months follow-up; advanced CKD subset
Rauen et al. 2018	Original RCT: Supportive Care + Immunosuppression (Steroids if baseline eGFR≥60; Steroids + Cyclophosphamide/Azathioprine if baseline eGFR 30-59) for 6 months or 3 years	Original RCT: Supportive Care alone	1.1 g/d	~58	STOP-IgAN trial post-hoc subgroup analysis; 3 years follow-up
Shin et al. 2016	Cyclophosphamide (oral or IV) + usually Azathioprine/MMF maintenance + usually steroids (concurrent)	Conservative management with RAS blockers only	UPCR ~1.8 g/g	~64	Retrospective cohort study; median 39 months follow-up

Abbreviations: APRIL, a proliferation-inducing ligand; AZA, azathioprine; BAFF, B-cell activating factor; BID, twice daily; BLyS, B-lymphocyte stimulator; CKD, chronic kidney disease; CrCl, creatinine clearance; CTX, cyclophosphamide; eGFR, estimated glomerular filtration rate; GFR, glomerular filtration rate; HCQ, hydroxychloroquine; IS, immunosuppression; IV, intravenous; mAb, monoclonal antibody; MMF, mycophenolate mofetil; PO, per os (oral); Q8W, every 8 weeks; QD, once daily; RAS, renin-angiotensin system; RCT, randomized controlled trial; SC, subcutaneous; UACR, urine albumin-to-creatinine ratio; UPCR, urine protein-to-creatinine ratio; UPE, urine protein excretion.

Supplementary Table S2: Meta-Regression of Factors Associated with Treatment Efficacy.

Outcome Measure	Covariate	Regression Coefficient (β)	Standard Error	P-value	R^2 Analog
**Proteinuria Reduction (%)**	Baseline Proteinuria (g/g or g/d)	5.31	2.17	0.023	0.31
	Baseline eGFR (mL/min/1.73m²)	0.11	0.18	0.551	0.02
	Study Duration (months)	0.42	0.21	0.049	0.24
	Mean Age (years)	-1.52	0.83	0.078	0.19
	Asian Ethnicity (%)	0.18	0.08	0.038	0.27
	Publication Year	0.89	0.41	0.041	0.25
	Concomitant RAS Blockade (%)	0.08	0.11	0.481	0.03
**eGFR Preservation (mL/min/1.73m²/year)**	Baseline Proteinuria (g/g or g/d)	1.12	0.54	0.046	0.28
	Baseline eGFR (mL/min/1.73m²)	0.06	0.04	0.142	0.15
	Study Duration (months)	0.15	0.05	0.006	0.39
	Mean Age (years)	-0.22	0.11	0.053	0.25
	Asian Ethnicity (%)	0.05	0.02	0.024	0.32
	Publication Year	0.19	0.10	0.071	0.20
	Concomitant RAS Blockade (%)	0.03	0.03	0.352	0.06
**Hard Kidney Outcomes (log HR)**	Baseline Proteinuria (g/g or g/d)	-0.29	0.12	0.032	0.38
	Baseline eGFR (mL/min/1.73m²)	0.01	0.01	0.283	0.10
	Study Duration (months)	-0.02	0.01	0.017	0.43
	Mean Age (years)	0.04	0.03	0.165	0.16
	Asian Ethnicity (%)	-0.01	0.00	0.041	0.35
	Publication Year	-0.02	0.03	0.476	0.04
	Concomitant RAS Blockade (%)	-0.00	0.01	0.611	0.02
**Serious Adverse Events (log RR)**	Treatment Category†	—	—	0.005	0.61
	Mean Age (years)	0.03	0.02	0.152	0.14
	Study Duration (months)	0.01	0.01	0.089	0.19
	Baseline eGFR (mL/min/1.73m²)	-0.01	0.01	0.121	0.16
	Asian Ethnicity (%)	0.01	0.00	0.008	0.41

Notes: †Treatment Category analyzed as a categorical variable with "Targeted-Release Corticosteroids" as reference group.

Supplementary Table S3: Risk of Bias Assessment for The Included Studies.

Study	Study Design	Selection Bias	Performance Bias	Detection Bias	Attrition Bias	Reporting Bias	Other Bias†	Overall Risk	Assessment Tool
**Perkovic et al. 2024**	RCT, Phase 3 (APPLAUSE-IgAN)	Low	Low	Low	Low	Low	Low	Low	RoB 2
**Kim et al. 2024**	RCT, Phase 3 (Reduced-dose TESTING)	Low	Low	Low	Low	Low	Low	Low	RoB 2
**Mathur et al. 2024**	RCT, Phase 2 (ENVISION)	Low	Low	Low	Low	Moderate	Low	Low/Moderate	RoB 2
**Barratt et al. 2023**	RCT, Phase 3 (NefIgArd Part A)	Low	Low	Low	Low	Low	Low	Low	RoB 2
**Zhang et al. 2024**	RCT, Phase 2 (Adaptive design)	Moderate	Low	Low	Low	Moderate	Low	Moderate	RoB 2
**Lv et al. 2022**	RCT, Phase 2/3 (Full & Reduced-dose TESTING)	Low	Low	Low	Low	Moderate	Low	Moderate	RoB 2
**Lafayette et al. 2024 (SANCTUARY - Ravulizumab)**	RCT, Phase 2 (SANCTUARY)	Low	Low	Low	Low	Low	Low	Low	RoB 2
**Lafayette et al. 2024 (ORIGIN - Atacicept)**	RCT, Phase 2b (ORIGIN)	Low	Low	Low	Low	Moderate	Low	Moderate	RoB 2
**Barratt et al. 2023 (OLE)**	Open-label Extension (OLE) of Phase 3 NefIgArd Part A	Moderate	High	Moderate	Low	Low	Moderate	Moderate	Modified RoB
**Fellström et al. 2017**	RCT, Phase 2b (NEFIGAN)	Low	Low	Low	Low	Low	Low	Low	RoB 2
**Rauen et al. 2020**	Retrospective Long-term Follow-up of RCT (STOP-IgAN 10yr)	Moderate	Moderate	Moderate	Moderate	Moderates	Moderate	Moderate	ROBINS-I
**Ma et al. 2021**	Retrospective Cohort (Propensity Score Matched Subset)	High	Moderate	Moderate	Moderate	Moderate	High	High	ROBINS-I
**Yang et al. 2019**	Case-Control (Propensity Score Matched)	High	Moderate	Moderate	Low	Moderate	High	High	ROBINS-I
**Liu et al. 2019**	RCT, Phase 2	Low	Low	Low	Low	Low	Low	Low	RoB 2
**Rauen et al. 2018**	Post-hoc Subgroup analysis of RCT (STOP-IgAN)	High	Moderate	Low	Low	High	High	High	RoB 2
**Lv et al. 2017**	RCT, Phase 2/3 (Full-dose TESTING - stopped early)	Low	Low	Low	Low	High	Moderate	High	RoB 2
**Shin et al. 2016**	Retrospective cohort	High	High	Moderate	Moderate	Moderate	High	High	ROBINS-I
**Kim et al. 2013**	RCT	Moderate	Low	Low	Low	Moderate	Low	Moderate	RoB 2
**Frisch et al. 2005**	RCT (stopped early)	Moderate	Low	Low	Moderate	Moderate	Moderate	Moderate	RoB 2
**Maes et al. 2004**	RCT	Moderate	Low	Low	Low	Low	Moderate	Moderate	RoB 2
**Pozzi et al. 2004**	Long-term Follow-up of RCT	Moderate	High	Moderate	Moderate	Moderate	Low	High	RoB 2
**Manno et al. 2009**	Long-term Follow-up of RCT	Moderate	High	Moderate	Moderate	Moderate	Low	High	RoB 2
**Manno et al. 2009 (Pilot)**	RCT Pilot (subset/early phase of Manno et al. 2009 main trial)	High	High	Moderate	Low	Moderate	High	High	RoB 2
**Pozzi et al. 1999**	RCT	Moderate	High	Moderate	Low	Low	Low	High	RoB 2
**Lv et al. 2009**	RCT	Moderate	High	Moderate	Low	Low	Low	High	RoB 2

Notes: †Other Bias: Includes funding source bias, baseline imbalances, confounding factors in non-randomized studies.

Supplementary Table S4: Publication Bias Assessment and Sensitivity Analyses.

Outcome	Treatment Category	Original Effect Size [95% CI]	Publication Bias Assessment	Influence Analysis	Risk of Bias Sensitivity Analysis
**Proteinuria Reduction**	All treatments	-28.4% [-33.9, -22.9]	Egger's p=0.089, Begg's p=0.145, Failsafe N=312	Range: -27.1% to -30.2%	Low-risk only: -31.5% [-37.6, -25.4] (7/25 studies)
	Complement Pathway Inhibitors	-31.2% [-38.1, -24.3]	Egger's p=0.627, Failsafe N=28	Range: -29.7% to -34.1%, Most influential: Perkovic 2024	Low-risk only: -32.4% [-40.3, -24.5] (2/3 studies)
	Targeted-Release Corticosteroids	-30.9% [-37.8, -24.0]	Egger's p=0.702, Failsafe N=24	Range: -28.5% to -32.7%, Most influential: Barratt 2023	Low-risk only: -28.6% [-37.5, -19.7] (2/3 studies)
	B-cell/Plasma Cell Targeted	-34.0% [-45.7, -22.3]	Egger's p=0.433, Failsafe N=34	Range: -31.2% to -36.4%, Most influential: Sibeprenlimab study	Low-risk only: -39.0% [-48.3, -29.7] (1/3 studies)
	Systemic Corticosteroids	-25.5% [-35.0, -16.0]	Egger's p=0.041*, Failsafe N=84	Range: -21.8% to -27.3%, Most influential: Lv 2017	Low-risk only: -24.3% [-35.1, -13.5] (1/6 studies)
	Antimalarials	-21.9% [-68.4, 24.6]	Not calculable, Failsafe N=2	Range: -58.4% to +14.4%, Most influential: Liu 2019	Low-risk only: -58.4% [-73.5, -43.3] (1/2 studies)
	Systemic Corticosteroids + ACEi	-35.0% [-48.4, -21.6]	Egger's p=0.238, Failsafe N=37	Range: -31.2% to -38.5%, Most influential: Lv 2009	Non-high risk only: Not available (0/3 studies)
**eGFR Preservation** (mL/min/1.73m²/year)	All treatments	+2.7 [1.8, 3.6]	Egger's p=0.132, Failsafe N=218	Range: +2.3 to +3.2	Low-risk only: +4.3 [3.1, 5.5] (5/18 studies)
	Complement Pathway Inhibitors	+5.8 [2.4, 9.2]	Not calculable, Failsafe N=8	Insufficient studies for analysis	Low-risk only: +6.7 [2.1, 11.3] (1/2 studies)
	Targeted-Release Corticosteroids	+4.2 [2.5, 5.9]	Egger's p=0.895, Failsafe N=22	Range: +3.8 to +4.5, Most influential: Barratt 2023	Low-risk only: +3.9 [1.5, 6.3] (2/3 studies)
	B-cell/Plasma Cell Targeted	+5.2 [3.1, 7.3]	Not calculable, Failsafe N=9	Insufficient studies for analysis	Low-risk only: +5.8 [3.2, 8.4] (1/2 studies)
	Systemic Corticosteroids	+2.6 [1.0, 4.2]	Egger's p=0.037*, Failsafe N=48	Range: +2.1 to +3.1, Most influential: Lv 2017	Low-risk only: +2.3 [0.2, 4.4] (1/4 studies)
	Antimalarials	+0.2 [-1.5, 1.9]	Not calculable	Insufficient studies for analysis	Low-risk only: +0.2 [-1.5, 1.9] (1/2 studies)
	Systemic Corticosteroids + ACEi	+4.1 [2.2, 6.0]	Egger's p=0.145, Failsafe N=31	Range: +2.9 to +4.9, Most influential: Manno 2009	Non-high risk only: Not available (0/3 studies)
	Systemic Corticosteroid + Cytotoxic Agent	+0.5 [-1.8, 2.8]	Not calculable	Range: -1.2 to +8.0, Most influential: Ma 2020	Non-high risk only: Not available (0/3 studies)
**Hard Kidney Outcomes**	All treatments	HR 0.42 [0.31, 0.57]	Egger's p=0.210, Failsafe N=79	Range: HR 0.36 to 0.47	Low-risk only: HR 0.33 [0.21, 0.52] (2/9 studies)
	Systemic Corticosteroids	HR 0.37 [0.26, 0.52]	Egger's p=0.621, Failsafe N=42	Range: HR 0.32 to 0.43, Most influential: Kim 2024	Low-risk only: HR 0.24 [0.10, 0.58] (1/3 studies)
	Systemic Corticosteroids + ACEi	RR 0.19 [0.07, 0.51]	Not calculable, Failsafe N=12	Range: RR 0.15 to 0.23, Most influential: Manno 2009	Non-high risk only: Not available (0/2 studies)
	Systemic Corticosteroid + Cytotoxic Agent	HR 0.77 [0.46, 1.28]	Not calculable, Failsafe N=4	Range: HR 0.35 to 1.20, Most influential: STOP-IgAN	Non-high risk only: Not available (0/2 studies)
	Classic Pulse Steroid Regimen	RR 0.42 [0.20, 0.88]	Not calculable	Single study	Non-high risk only: Not available (0/1 studies)
	Antimetabolites	RR 1.66 [0.63, 4.36]	Not calculable	Range: RR 1.15 to 3.20, Most influential: Frisch 2005	Non-high risk only: Not available (0/2 studies)

Abbreviations: ACEi: Angiotensin-Converting Enzyme inhibitor; CI: Confidence Interval; eGFR: estimated Glomerular Filtration Rate; HR: Hazard Ratio; RR: Risk Ratio. **Note:** *Statistically significant (P-value<0.05), suggesting possible publication bias for this specific analysis.
